# Ultraviolet A phototest positivity is associated with higher free erythrocyte protoporphyrin IX concentration and lower transferrin saturation values in erythropoietic protoporphyria

**DOI:** 10.1111/phpp.12727

**Published:** 2021-08-30

**Authors:** Giovanni Genovese, Carlo Alberto Maronese, Chiara Moltrasio, Roberta Piccinno, Dario Antonio Marletta, Giacomo De Luca, Giovanna Graziadei, Francesca Granata, Elena Di Pierro, Maria Domenica Cappellini, Angelo Valerio Marzano

**Affiliations:** ^1^ Dermatology Unit Fondazione IRCCS Ca' Granda Ospedale Maggiore Policlinico Milan Italy; ^2^ Department of Pathophysiology and Transplantation Università degli Studi di Milano Milan Italy; ^3^ Department of Medical Surgical and Health Sciences University of Trieste Trieste Italy; ^4^ General Medicine Unit Rare Disease Center Fondazione IRCCS Ca' Granda Ospedale Maggiore Policlinico Milan Italy; ^5^ Department of Clinical Science and Community Università degli Studi di Milano Milan Italy

**Keywords:** erythropoietic protoporphyria, photosensitivity, phototesting, porphyria, protoporphyrin IX, UVA phototesting

## Abstract

**Background:**

Erythropoietic protoporphyria (EPP) is a rare disorder of heme biosynthesis hallmarked by early‐onset photosensitivity and mainly due to defective ferrochelatase activity leading to increased erythrocyte protoporphyrin IX (PPIX) levels. Evidence regarding the relationship between erythrocyte PPIX concentration and photosensitivity is limited.

**Methods:**

To investigate the relationship between free erythrocyte PPIX (FEP) concentration; routine laboratory tests, particularly iron metabolism biomarkers; and ultraviolet (UV) A/visible light phototesting findings, 20 genetically confirmed EPP and one XLPP treatment‐naive patients were included in our study. They underwent UVA and visible light phototesting. On the same day, blood samples were collected for measurement of FEP, serum iron, transferrin, transferrin saturation, and ferritin, 25‐hydroxyvitamin D, and liver enzyme levels.

**Results:**

Median FEP concentration at the time of phototesting was 57.50 (IQR: 34.58‐102.70) μg/g of Hb. UVA and visible light phototesting were positive in 9 (42.9%) and 8 (38.1%) patients, respectively. Median FEP concentration was significantly higher in UVA phototest–positive patients than in those negative (64.37 [IQR: 57.45‐121.82] vs 45.35 [IQR: 24.53‐74.61] μg/g of Hb, respectively; *P* = .04486). Similarly, UVA photosensitive individuals had significantly lower median serum iron levels (61.5 [IQR: 33.5‐84] μg/dL vs 109 [IQR: 63.25‐154] μg/dL, respectively; *P* = .01862) and transferrin saturation values (15.005 [IQR: 7.0775‐18.41] % vs 29.645 [IQR: 17.8225‐34.3575] %; *P* = .0109) than those negative.

**Conclusions:**

Our study demonstrates that UVA phototest positivity is associated with higher FEP concentration and lower transferrin saturation and serum iron concentration in EPP.

## 1 INTRODUCTION

Erythropoietic protoporphyria (EPP) is a rare metabolic disease of heme biosynthesis characterized by cutaneous manifestations, including acute photosensitivity associated with painful erythematous‐edematous changes, lichenification of chronically sun‐exposed areas, and grooving around the lips; occasional microcytic anemia; and potential development of cholestatic hepatopathy.^1^ With 0.12 (0.10‐0.15) new cases per year per million inhabitants in Europe and 0.07 (0.04‐0.12) in Italy, it is the second highest incidence of cutaneous porphyria.^2^

EPP may be caused either by inborn or acquired impairment of the activity of ferrochelatase, the last enzyme in the heme biosynthetic pathway, resulting in elevated protoporphyrin IX (PPIX) levels both in erythrocytes and plasma. Loss‐of‐function (LOF) mutations of the ferrochelatase (*FECH*) gene co‐occurring in trans with a common hypomorphic variant (rs2272783; c. 315‐48C) account for most inherited EPP.^3^

PPIX excitation spectrum shows a peak in the wavelength region between late ultraviolet (UV) A and visible light, with its maximum at 410 nm. Properties of the accumulating porphyrins influence the anatomic site of the phototoxic reaction. Indeed, the lipophilic nature of PPIX determines its localization within cellular membranes, such as those of endothelium and erythrocytes, thus explaining the acute pain, erythema, and edema in the absence of blistering.^4^ Great inter‐ and intraindividual variabilities have been documented in light tolerance of EPP patients. According to a large survey,^5^ median times for onset of symptoms after sun exposure, onset of signs (erythema, edema) and resolution of symptoms are 20 minutes, 6 hours and 3 days, respectively. Moreover, increased photosensitivity can be observed in the days after sunlight exposure.^6^ This phenomenon, known as photopriming, may be the result of leaking blood vessels transferring increasing amounts of PPIX into the skin after an initial phototoxic injury.^7^, ^8^, ^9^

Data concerning the correlation between PPIX levels and photosensitivity are inconclusive, and only three studies used phototests for evaluating photosensitivity in EPP patients including a total of 31 individuals.^8^, ^10^, ^11^ In current clinical practice, severity assessment of EPP patients is clinical, use of phototesting is marginal, and validated laboratory markers capable of reliably predicting photosensitivity are lacking. In the present study, we focused on standard phototesting to better simulate natural sunlight exposure and avoid confirmation bias from testing in the Soret band, exclusively.

The primary endpoint of our study conducted in a single‐center cohort of EPP patients was to assess whether phototest‐positive and ‐negative individuals have different levels of FEP, serum iron, serum ferritin, transferrin, transferrin saturation, serum 25‐hydroxyvitamin D (25OHD), aspartate transaminase, alanine transaminase, and gamma‐glutamyl transferase. The secondary endpoint was to explore the correlation between FEP concentration, patient age, and iron metabolism biomarkers.

## 2 METHODS

### 2.1 Patients

This was a cross‐sectional study conducted at the Dermatology Unit and at the Rare Disease Center of the Fondazione IRCCS Ca' Granda Ospedale Maggiore Policlinico, Milan. The study was conducted in accordance with the Declaration of Helsinki and approved by the Institutional Review Board of the Ethical Committee (number 133_2018). All patients provided written informed consent before study initiation.

Patients were recruited between September 2018 and February 2019. Eligibility criteria were as follows: (a) age older than 18 years and (b) genetically proven diagnosis of EPP. Individuals who had been previously treated with afamelanotide or UVB hardening phototherapy and pregnant patients were ruled out. Moreover, patients taking any medications and/or dietary supplements (eg, vitamin D, iron, and beta‐carotene) to increase their tolerance to sunlight and either systemic or topical photosensitizing drug in the 24 weeks preceding the test were excluded.

### 2.2 Study protocol

In March 2019, each patient attended the Phototherapy Outpatient Service of our Dermatology Unit to undergo UVA and visible light phototesting. To prevent the potential biases ensuing from the priming phenomenon, phototesting was performed on non‐sun‐exposed areas, and patients were asked to avoid sun exposure in the preceding week.

On the same day, blood samples were collected for measurement of FEP concentration, serum iron, serum transferrin, serum ferritin, 25OHD, and liver function tests. Transferrin saturation was calculated using the following formula: (iron (μg/dL)/transferrin (mg/dl)) × 71.24.

UVA phototesting was performed on an 8 × 4‐cm area on left gluteal skin. Upper gluteal skin represents a classical site for the execution of phototesting and was chosen for both practical and ethical reasons.

Initial irradiation time was 15 minutes and then half of the said area was irradiated for 5 additional minutes. A photochemotherapy device (Waldmann PUVA 3001) was employed for UVA delivery, with an emission spectrum peaking at around 365 and 405 nm. The delivered UVA dosage was 7 J/cm^2^ for the first 15 minutes and 9 J/cm^2^ for the entire duration of the phototest.

Visible light phototesting was carried out with a standard halogen lamp slide projector (NOVAMAT 820, 150 W; emission spectrum 400‐1000 nm) on a 10 × 10‐cm area on right gluteal skin. Initial irradiation lasted 15 minutes and then half of the area was irradiated for 5 additional minutes. The delivered visible light dosage was 1.79 J/cm^2^ for the first 15 minutes and 2.39 J/cm^2^ for the entire duration of the phototest.

The distance between skin and light source was 30 cm for visible light phototesting and 20 cm for UVA phototesting. Although both artificial sources for phototesting produce heat, they were endowed with a ventilation system to reduce the heating effect on the skin.

Results of UVA and visible light phototesting were read immediately and after 15 minutes. Phototesting positivity was based on the latter readings. A cautionary additional reading was performed after 24 hours. Both objective and subjective clinical variables, such as erythema, edema, and burning or itching sensation, were recorded. The severity of each manifestation was assessed using a three‐level scoring system (absent, 0; mild, +; intense, ++) adapted from the European Dermatology Guideline for the photodermatoses.^12^ Finally, phototest positivity was defined based on the presence of at least one sign or symptom.

### 2.3 Statistical analysis

The normality of distribution of continuous variables was assessed by the Shapiro‐Wilk test. Continuous variables with normal distribution were presented as mean ± standard deviation (SD); non‐normal variables were reported as median (interquartile range [IQR]).

The Kruskal‐Wallis test was used for comparison between groups. Pearson's *r* was used to assess the correlation between continuous variables. A *P*‐value <.05 was considered statistically significant. Statistical software SAS (release 9.4; SAS Institute, Inc) was used to perform all the statistical analyses.

## 3 RESULTS

Age, genetic data, laboratory findings, and phototesting results of the 20 EPP and 1 XLPP patients included in our study are summarized in Tables 1 and 2. Twelve patients were male, with a male‐to‐female ratio of 1.30. Median age at phototesting was 39.26 (28.51‐49.83) (range: 20.40‐56.40) years. All subjects were phototype III on the Fitzpatrick scale. Median FEP concentration at the time of phototesting was 57.50 (IQR: 34.58‐102.70) μg/g of Hb (reference range: 0.00‐3.00 μg/g of Hb). No statistically significant correlation was found between FEP concentration and patient age (*r* = .366; *P* = .103; *R* ^2^ = 0.134). FEP concentration at the time of phototesting did not differ significantly between male and female individuals, even after excluding a female outlier with particularly high FEP concentration (84.21 [IQR:34.04‐111.18] vs 44.43 [IQR:30.43‐54.55] μg/g of Hb, *P* = .0538).

**TABLE 1:** Patients' characteristics including sex, age, genetics, free erythrocyte protoporphyrin IX concentration, and phototesting results

	Sex, age (y)	Genetic changes	Reference	FEP concentration (μg/g of Hb) (reference range: 0.00‐3.00)	UVA phototesting		Visible light phototesting	
					Signs	Symptoms	Signs	Symptoms
1	M, 24	c.[215dupT];[315‐48T>C]	16	10.75	0	0	0	0
2	F, 44	c.[901_902delTG];[315‐48T>C]	17	13.87	0	0	0	0
3	M, 20	c.[67+5 G>A];[315‐48T>C]	18	23.97	0	0	0	0
4	M, 56	c.[464‐1169 A>C];[315‐48T>C]	19	26.22	0	0	0	0
5	F, 39	c.[1706_1709delAGTG];[=]^a^	20	26.29	0	Burning sensation +	0	Burning sensation ++
6	F, 31	c.[215dupT];[315‐48T>C]	16	42.86	0	0	0	Burning sensation ++
7	F, 37	c.[1‐251G>C;194+4350_463+1197del5577]; [315‐48T>C]	21	44.06	0	Burning sensation ++	0	Burning sensation +
8	F, 26	c.[215dupT];[315‐48T>C]	16	44.80	0	0	0	0
9	F, 53	c.[464‐1169A>C]; [315‐48T>C]	19	45.89	0	0	0	0
10	F, 26	c.[1080_1081delTG];[315‐48T>C]	3	57.43	0	Burning sensation ++	0	Burning sensation ++
11	M, 23	c.[67+5 G>A];[315‐48T>C]	18	57.50	0	Burning sensation +	0	0
12	F, 48	c.[215dupT];[315‐48T>C]	16	59.38	0	0	0	0
13	M, 51	c.[1‐251G>C;194+4350_463+1197del5577]; [315‐48T>C]	21	64.99	0	0	0	Burning sensation +
14	M, 33	c.[215dupT];[315‐48T>C]	16	77.81	0	0	0	0
15	M, 45	c.[215dupT];[315‐48T>C]	16	90.60	Erythema +	Burning sensation +	Erythema +	0
16	M, 50	c.[464‐1169 A>C];[315‐48T>C]	19	97.87	0	0	0	0
17	M, 43	c.[901_902delTG];[315‐48T>C]	17	107.52	Erythema +	Burning sensation +	0	Burning sensation ++
18	M, 34	c.[1‐251G>C;194+4350_463+1197del5577]; [315‐48T>C]	21	112.41	0	Itch +	0	0
19	M, 39	c.[215dupT]; [315‐48T>C]	16	124.95	Erythema ++, oedema +	Burning sensation ++	Erythema +, oedema +	Burning sensation ++
20	M, 52	c.[215dupT]; [315‐48T>C]	16	133.38	0	0	0	0
21	F, 50	c.[901_902delTG]; [315‐48T>C]	17	215.11	0	Burning sensation ++	0	0

Abbreviations: FEP, free erythrocyte protoporphyrin IX; Hb, hemoglobin.

^a^ Genotype referred to the *ALAS2* gene.

**TABLE 2:** Demographic, clinical, and laboratory features of the 21 patients included in the study

Age at phototesting, median (IQR)		39.26 (28.51‐49.83)
Males, n (%)		12 (57.14)
Females, n (%)		9 (42.86)
Skin symptoms, n (%)	Burning sensation	10 (47.62)
	Itch	1 (4.76)
Laboratory findings at the time of phototesting	FEP concentration, μg/g of Hb [median (IQR)]; reference range: 0.00‐3.00	57.5 (34.58‐102.70)
	Serum iron levels, μg/dL [median (IQR)]; reference range: 37‐145	88 (57‐127)
	Serum transferrin, mg/dL [median (IQR)]; reference range: 200‐360	297 (267.50‐330)
	Transferrin saturation, % [median (IQR)]; reference range: 15‐50	18.94 (14.38‐31.26)
	Serum ferritin levels, μg/L [median (IQR)]; reference range: 15‐150	37 (14‐67.50)
	Serum 25OHD levels, μg/L [median (IQR)]; normal values: >20	15.80 (9.80‐23.40)
	AST, U/L [median (IQR)]; reference range: 10‐33	24 (20‐34)
	ALT, U/L [median (IQR)]; reference range: 6‐41	30 (19.50‐41.50)
	GGT, U/L [median (IQR)]; reference range: 5‐36	19 (11‐25)

Abbreviations: 25OHD, 25 hydroxyvitamin D; ALT, alanine aminotransferase; AST, aspartate aminotransferase; FEP, free erythrocyte protoporphyrin IX; GGT, gamma‐glutamyl transferase; IQR, interquartile range.

The median serum iron level in our EPP cohort was 88 (IQR: 57‐127) μg/dL (reference range: 37‐145 μg/dL). The median serum transferrin level was 297 (IQR: 267.50‐330) mg/dL (reference range: 200‐360 mg/dL). The median transferrin saturation value in our cohort was 18.94 (IQR: 14.38‐31.26) % (reference range: 15%‐50%). Median serum ferritin concentration was 37 (IQR:14‐67.50) μg/L (reference range: 15‐150 μg/L). Liver function tests were within normal ranges (AST: 10‐33 U/L; ALT: 6‐41 U/L; GGT: 5‐36 U/L) in all tested individuals with only two exceptions. Median values for AST, ALT, and GGT were 24 (IQR: 20‐34) U/L, 30 (IQR: 19.50‐41.50) U/L, and 19 (IQR: 11‐25) U/L, respectively.

Overall, 11 (52.38%) patients had at least one positive phototest. Six individuals were positive to both UVA and visible light phototesting, of whom one had XLPP. Their FEP concentrations ranged widely (26.29‐124.95 μg/g of Hb). Symptoms and signs determined by UVA or visible light exposure disappeared completely at a 24‐hour reading.

Nine (42.86%) individuals had positive UVA phototesting. Among EPP patients with a positive UVA phototest, nine complained of either burning sensation (n = 8; 38.01%) or itch (n = 1; 4.76%). Erythema manifested in three (14.29%) cases at the end of the procedure, and accompanying edema was present only in one individual (4.76%).

Positive visible light phototesting was noted in 8 (38.10%) patients. Seven (33.33%) of those with a positive visible light phototest complained of a burning sensation. Erythema presented in two (9.52%) cases, with accompanying edema in one of them (4.76%).

The following analyses refer to the 20 patients with FECH gene‐related EPP exclusively.

FEP concentration was significantly higher in patients with positive UVA phototest than in those with negative UVA phototest (64.37 [IQR: 57.45‐121.82] vs 45.35 [IQR: 24.53‐74.61] μg/g of Hb, respectively; *P* = .0449). No statistically significant difference was documented between patients with positive and negative visible light phototesting in terms of FEP concentration (64.99 [IQR: 44.06‐107.52]) vs 57.5 [IQR: 25.10‐105.14] μg/g of Hb, respectively; *P* = .6065).

A weak, yet statistically significant correlation was documented between FEP concentration and serum transferrin level (*r* = .46; *P* = .0411; *R* ^2^ = 0.2119), whereas no significant correlation was recorded between FEP concentration and serum iron level (*r* = −.10; *P* = .6780; *R* ^2^ = 0.0099), FEP concentration and transferrin saturation (*r* = −.17; *P* = .4710; *R* ^2^ = 0.0293), or FEP concentration and ferritin (*r* = −.02; *P* = .9323; *R* ^2^ = 0.0004). UVA photosensitive individuals had significantly lower serum iron levels than those with a negative UVA phototest (61.50 [IQR: 33.50‐84] μg/dL vs 109 [IQR: 63.25‐154] μg/dL, respectively; *P* = .0186). Patients manifesting visible light photosensitivity and those with a negative test did not differ in terms of serum iron levels (60 [IQR: 38‐88] μg/dL vs 100 [IQR: 65‐146] μg/dL, respectively; *P* = .0746).

Serum transferrin did not differ between UVA photosensitive individuals and those with a negative UVA phototest (310 [IQR: 278‐347.50] mg/dL vs 275.5 [IQR: 256.25‐322.50] mg/dL, respectively; *P* = .1228). Visible light phototest–positive and –negative individuals did not differ significantly in terms of serum transferrin levels (290 [IQR: 267‐331] mg/dL vs 297 [IQR: 265‐330.50] mg/dL, respectively; *P* = .7815).

UVA phototest–positive EPP patients demonstrated significantly lower transferrin saturation values than UVA phototest‐negative subjects (15.01 [IQR: 7.08‐18.41] % vs 29.65 [IQR: 17.82‐34.36] %; *P* = .0109). Visible light phototest–positive vs –negative individuals did not differ in terms of transferrin saturation (16.82 [IQR: 7.67‐18.94] % vs 26 [IQR: 14.97‐33.37] %; *P* = .1655).

No statistically significant differences in terms of serum ferritin concentrations were found between UVA phototest–positive and –negative patients (16.50 [IQR: 14‐56.75] vs 42.5 [IQR: 15.75‐89.5] μg/L, respectively; *P* = .3545) and visible light test–positive and –negative subjects (17 [IQR: 14‐77] vs 37 [IQR: 12.5‐50.5] μg/L; *P* = .9368). No statistically significant differences in terms of 25OHD levels (normal values: >20) were documented between the UVA phototest–positive and –negative subjects (15.80 [IQR: 12.50‐25.30] vs 17.55 [IQR: 9.65‐21.78]; *P* = .6726) and the visible light test–positive and –negative subjects (12.30 [IQR: 7.70‐26.58] vs 19.70 [IQR: 13.80‐22‐75]; *P* = .3805).

ALT values proved to be higher in UVA photosensitive individuals than those with a negative UVA phototest (36.50 [IQR: 31‐48.75] vs 23 [IQR: 18.25‐30.75] *P* = .0253). Pearson's *r* for the correlation between ALT levels and FEP concentrations was 0.24 but did not reach statistical significance (*P* = .3117). No other statistically significant difference in terms of liver function tests was recorded between different phototest response groups (Table 3).

**TABLE 3:** Laboratory findings in EPP patients (n = 20) at the time of phototesting stratified by the result of UVA phototesting and visible light phototesting

Laboratory findings at the time of phototesting	UVA phototesting		*P* value	Visible light phototesting		*P* value
	Positive (n = 8)	Negative (n = 12)		Positive (n = 7)	Negative (n = 13)	
FEP concentration, μg/g of Hb [median (IQR)]	64.37 (57.45‐121.82)	45.35 (24.53‐74.61)	.**0449**	64.99 (44.06‐107.52)	57.50 (25.10‐105.14)	.6065
Serum iron levels, μg/dL [median (IQR)]	61.50 (33.50‐84)	109 (63.25‐154)	.**0186**	60 (38‐88)	100 (65‐146)	.0746
Serum transferrin, mg/dL [median (IQR)]	310 (278‐347.50)	275.50 (256.25‐322.50)	.1228	290 (267‐331)	297 (265‐330.50)	.7815
Transferrin saturation, % [median (IQR)]	15.01 (7.08‐18.41)	29.645 (17.82‐34.36)	.**0109**	16.82 (7.67‐18.94)	26 (14.97‐33.37)	.1655
Serum ferritin levels, μg/L [median (IQR)]	16.50 (14‐56.75)	42.50 (15.75‐89.50)	.3545	17 (14‐77)	37 (12.50‐50.50)	.9368
Serum 25OHD levels, μg/L [median (IQR)]	15.80 (12.50‐25.30)	17.55 (9.65‐21.78)	.6726	12.30 (7.70‐26.58)	19.70 (13.80‐22‐75)	.3805
AST, U/L [median (IQR)]	35 (21‐63.75)	22.50 (20.25‐24.75)	.1325	24 (20‐39)	24 (20.50‐31.50)	.8121
ALT, U/L [median (IQR)]	36.50 (31‐48.75)	23 (18.25‐30.75)	.**0253**	45 (22‐50)	25 (19‐33)	.1223
GGT, U/L [median (IQR)]	21.50 (12.50‐35)	16.50 (10.25‐22.75)	.2318	23 (12‐35)	19 (10.50‐22.50)	.3029

Abbreviations: 25OHD, 25 hydroxyvitamin D; ALT, alanine aminotransferase; AST, aspartate aminotransferase; FEP, free erythrocyte protoporphyrin IX; GGT, gamma‐glutamyl transferase; IQR, interquartile range; SD, standard deviation; UVA, ultraviolet A.

Bold *P* values are statistically significant.

## 4 DISCUSSION

The dynamics of FEP concentration in EPP are quite complex. A retrospective observational study including 53 Danish EPP patients aged 0‐90 years demonstrated that the intraerythrocyte levels of PPIX increase with age up until adulthood and are higher in men.^13^ Consistently with the study by Heerfordt et al,^13^ no significant correlation between age and FEP concentration could be demonstrated in our cohort, which consisted entirely of adults.

Moreover, Heerfordt et al^13^ reported the presence of seasonal fluctuations in FEP concentration, which tend to decrease during summer. This is hypothesized to take place as a consequence of PPIX inactivation in dermal vessels during summer months. In order to standardize our data and avoid measurement bias related to the execution of phototests under different weather conditions, we decided to concentrate the measurements in a reduced time window of 1 month (March).

The clinical relevance of different erythrocyte PPIX levels in predicting cutaneous photosensitivity is not entirely clear. Although erythrocyte PPIX levels have been reported to be significantly related to photosensitivity,^8^ no convincing evidence of a simple correlation has been documented.^14^ Previous reports failed to demonstrate a correlation between total erythrocyte porphyrin levels and time to symptom onset.^5^ Heerfordt and Wulf^8^ found that skin PPIX was significantly associated with erythrocyte PPIX, skin erythema, and symptoms, namely, stinging or pain, during controlled illumination. However, another study by the same authors documented that increasing FEP concentration correlated neither with tolerable daily light dose nor with percentages of days with symptoms.^6^ Moreover, cutaneous ultra‐weak photoemissions, a by‐product of PPIX‐induced phototoxic reactions, were also shown to correlate with erythrocyte PPIX levels.^15^

In the present study, significantly higher FEP concentration was documented in individuals with a positive UVA phototest. Moreover, no statistically significant difference in terms of FEP concentration was found between patients with a positive visible light phototest and those with a negative one.

In our cohort, seven different *FECH* gene mutations^16^, ^17^, ^18^, ^19^, ^20^, ^21^, ^22^, ^23^ were detected in 20 patients, confirming the wide genetic heterogeneity already described in EPP.^3^, ^22^, ^23^ Although the c.215dupT mutation was found to be prevalent, accounting for 38% of patients, both FEP values and phototest positivity were highly variable among patients carrying the same mutation.

However, differential expression of erythrocyte membrane transporter ABCG2 could explain the lack of a linear relation between FEP concentration and photosensitivity. First described by Wang et al,^24^ ABCG2 is a potential mediator in EPP pathophysiology, modulating PPIX leakage from bloodstream to the skin. Speculatively, this may provide valuable insights into cases from our cohort exhibiting very high FEP concentrations but negative phototesting results.

Routine EPP assessment by means of phototesting remains limited and controversial.^25^ Mathews‐Roth et al^26^ highlighted the value of polychromatic light phototesting as an objective measure of EPP photosensitivity, especially when assessing treatment response. In our study, positivity of UVA phototesting, rather than polychromatic visible light, was associated with significantly higher FEP concentration in EPP patients.

Another noteworthy finding of our study was that both serum iron levels and transferrin saturation values were significantly lower in the group with positive UVA phototest than in the group with negative UVA phototest. Interestingly, transferrin saturation was shown to be lower in UVA but not visible light photosensitive patients. Erythropoiesis and iron metabolism are known to be altered in EPP,^27^ but to the best of our knowledge, the association between iron levels, transferrin saturation, and UVA photosensitivity has never been reported previously. Iron levels and expression of aminolevulinic acid synthase (ALAS) 2 – the first enzyme in the heme biosynthetic pathway – are known to behave as disease modifiers in EPP. Indeed, ALAS2 appears to be elevated in EPP patients, possibly as a result of an altered feedback mechanism, thereby contributing to PPIX accumulation downstream. Iron deprivation has been shown to hinder ALAS2 translation. Interestingly though, both improvement and aggravation of symptoms have been reported following oral iron supplementation in ferrochelatase‐deficient EPP.^28^, ^29^, ^30^, ^31^, ^32^ Improvements may have occurred in patients with previously undiagnosed XLPP. Indeed, in XLPP, iron supplementation sequesters PPIX and reverses liver damage and photosensitivity^33^, ^34^ by converting toxic PPIX into heme. Conversely, strong evidence confirms the benefits of mild anemia on photosensitivity symptoms in EPP patients.^34^

Of note, only weak, yet statistically significant, correlation was documented between FEP concentration and serum transferrin level. No meaningful link was traced between FEP concentration and other iron‐related parameters.

Curiously, slightly higher levels of ALT were measured in UVA photosensitive individuals than those with a negative UVA phototest. ALT levels were well within normal ranges in both groups, and none of the subjects had a history of hepatopathy. Although this finding could be linked to both disease severity and risk of future liver disease, its interpretation remains uncertain.

The main limitation of the present study is the scarce numerosity of our cohort mainly due to the rarity of EPP, and its major strength resides in the use of phototesting as objective means for the assessment of acute photosensitivity and related symptoms. Other noteworthy limitations include (a) the propensity of patients to be more aware of symptoms and to report them in a clinical setting, rather than in everyday life; (b) day‐to‐day variability of photosensitivity in EPP; (c) differences in photosensitivity at different body sites due to discrepancies in dermal thickness and vascular density^6^, ^35^; (d) the lack of 7‐hour readings,^36^ which were not performed due to practical reasons; (e) although UVA radiation accounts for more than 95% of total emissions of our UVA source, a small, negligible percentage of radiations was represented by blue light–skewed visible light.

In conclusion, the present study provides novel insights into the relationship between FEP concentration, laboratory findings, and phototesting results in EPP patients. Although higher FEP levels, lower serum iron concentrations, and transferrin saturation values were found in patients with positive UVA phototesting results, photosensitivity to both UVA and visible light was documented in the majority of those with at least a positive phototest result, indicating the viability of both techniques in the evaluation of EPP. Iron metabolism imbalance may be an epiphenomenon of disease severity, which also translates into higher photosensitivity. From a practical perspective, we believe that these findings may provide guidance in the management of the condition, counseling patients so that they may avoid unintended UVA exposure, especially if the laboratory examinations, such as low transferrin saturation, lower serum iron, and higher FEP levels, suggest greater proneness to UVA photosensitivity. Further research on larger samples will be required to confirm our findings.

## CONFLICT OF INTEREST

Giovanni Genovese, Carlo Alberto Maronese, Chiara Moltrasio, Roberta Piccinno, Dario Antonio Marletta, Giacomo De Luca, Giovanna Graziadei, Francesca Granata, Elena Di Pierro, Maria Domenica Cappellini, and Angelo Valerio Marzano declare that they have no conflict of interest.

## AUTHOR CONTRIBUTIONS

Giovanni Genovese and Carlo Alberto Maronese equally participated in data analysis and interpretation and drafting of the manuscript. Giacomo De Luca and Giovanna Graziadei participated in acquisition of clinical and laboratory data. Dario Antonio Marletta and Roberta Piccinno performed the phototests and participated in acquisition of data. Elena Di Pierro, Francesca Granata, and Chiara Moltrasio participated in acquisition and interpretation of genetic data. Angelo Valerio Marzano and Maria Domenica Cappellini participated in study concept and design and supervised the study. All authors critically revised the manuscript for important intellectual content and approved the final manuscript.

## ETHICS APPROVAL

The study was approved by the Institutional Review Board of the Ethical Committee (number: 133_2018) and conducted in accordance with the ethical standards of the responsible committee on human experimentation (institutional and national) and with the Helsinki Declaration of 1975, as revised in 2000.

## PATIENT CONSENT STATEMENT

Written informed consent was obtained from all patients for being included in the study.

## Data Availability

Anonymized data will be shared upon reasonable request from any qualified investigator for purposes of replicating procedures and results.
